# Supplementary material for: Optimized Multi-Epitope Norovirus Vaccines Induce Robust Humoral and Cellular Responses in Mice
Source: Vaccines (Basel). 2025 Dec 31;14(1):50. doi: 10.3390/vaccines14010050 (PMC12846433; doi:10.3390/vaccines14010050)
Supplement: Supplementary file 1 [file vaccines-14-00050-s001.zip › vaccines-3972555-supplementary.pdf]

Supplementary Materials: Figures and Tables

Table S1. Homology identification of protein with human genome.(>100 bit score and <30% percent sequence identity.

Protein Candidate	Human	Lactobacillus rhamnosus	Lactobacillus johnsonii	Lactobacillus casei
GI Polyprotein				Similar
GIV Polyprotein				
GI VP1				
GI VP2	No-Similarity	No-Similarity	No-Similarity	
GII VP1				No-Similarity
GII VP2				
GIV VP1				
GIV VP2				

Table S2. Antigenicity, allergenicity and transmembrane helix of remaining vaccine candidates.

Protein Candidate	Antigenicity	Allergenicity	Transmembrane helix
GII VP2	0.45	Yes	0
GIV VP2	0.51		0
GI VP1	0.47		0
GI VP2	0.49		0
GII VP1	0.55	No	0
GIV VP1	0.52		0

Table S3. Prediction and assessment of cytotoxic T-lymphocyte epitopes (CTL).

Epitopes	HLA Restriction	Percentile Rank	Allergenicity	Toxicity	Coverage	Hydropathicity
TYPGEQILF	HLA-A*24:02/HLA-C*04:01	0.01			30.76%	0.01
NIIDPWIMK	HLA-A*11:01	0.04			43.48%	0.22
TLEPIFIPV	HLA-A*02:01	0.31	No	Non-Toxin	14.62%	1.38
LMAGNAFTA	HLA-A*02:01	0.32			14.62%	1.03
VLMAGNAFT	HLA-A*02:01	0.37			14.62%	1.30
IFAAVPPHF	HLA-A*24:02	0.01		-	-	-
AVVSTRSPK	HLA-A*11:01	0.10	Yes	-	-	-
GLASSVFPT	HLA-A*02:01	0.17		-	-	-
RFDSWVNQF	HLA-A*24:02/HLA-C*04:01	0.01		-	-	-

Table S4. Prediction and assessment of helper T-lymphocyte epitopes (HTL).

Epitopes	Methods	Percentile Rank	Allergenicity	Toxicity	Coverage	Hydropathicity
PVAGGAIAA	DQA1*05:01	0.03	No	Non-Toxin	32.43%	1.50
FTAGKVIFA	DQA1*05:01	0.05				1.43
IIVDVRTLE	DRB1*03:01	0.04	Yes	-	-	-
LLRYVNPET	DRB1*15:01	0.07		-	-	-
LEPTKFTPI	DPA1*01:03/DPB1*04:01	0.10		-	-	-
AGGAIAAPL	DQA1*05:01/DRB1*03:01	0.19		-	-	-
IESGQSGPA	DQA1*05:01/DRB1*03:01	0.28		-	-	-

Table S5. Prediction and assessment of linear B-cell epitopes (LBL).

Epitopes	Methods	Toxicity	Allergenicity	Hydropathicity
FFRSYIPLKGGFGNTA	ANN	Non-Toxin	No	0.36
LKGGFGNTAI	DT			0.07
ELQGTTQLVTP	DT			-0.25
QGTTQLVTPN	DT			-0.66
HEGNDDNHK	DT			-3.13
AAFDPTEDVPAP	DT			-0.30
IESGQSGPANRS	DT			-1.12
RNRSIESGQSGPAN	DT			-1.54
NHKWHMTVTSPNGAAF	ANN			-0.62

Table S6. Antigenicity, allergenicity and various physicochemical properties of candidates.

Vaccine Candidate	Antigenicity	Allergenicity	Solubility	Estimated half-life	Instability index	Aliphatic index	GRAVY
7PC12345H12B123	0.5091	No	0.640	30h Mammalian reticulocytes in vitro >20h Yeast in vivo >10h E.coli in vivo	31.20	75.55	-0.173
3PCHB7	0.5082	No	0.675	1.1h Mammalian reticulocytes in vitro 3min Yeast in vivo 2min E.coli in vivo	-	-	-
3PCBH7	0.4572	-	-	-	-	-	-
3PHCB7	0.4636	-	-	-	-	-	-
3PHBC7	0.4834	-	-	-	-	-	-
3PBCH7	0.4806	-	-	-	-	-	-
3PBHC7	0.4447	-	-	-	-	-	-
7PCHB3	0.5418	No	0.675	30h Mammalian reticulocytes in vitro >20h Yeast in vivo >10h E.coli in vivo	34.74	73.29	-0.402
7PCBH3	0.4908	-	-	-	-	-	-
7PHCB3	0.4972	-	-	-	-	-	-
7PHBC3	0.5170	No	0.675	30h Mammalian reticulocytes in vitro >20h Yeast in vivo >10h E.coli in vivo	35.13	73.29	-0.402
7PBCH3	0.5142	No	0.675	30h Mammalian reticulocytes in vitro >20h Yeast in vivo >10h E.coli in vivo	34.74	73.29	-0.402
7PBHC3	0.4782	-	-	-	-	-	-
3PCCCCCC7	0.5617	No	0.591	1.1h Mammalian reticulocytes in vitro 3min Yeast in vivo 2min E.coli in vivo	-	-	-
7PCCCCCC3	0.5913	No	0.591	30h Mammalian reticulocytes in vitro >20h Yeast in vivo >10h E.coli in vivo	36.07	90.86	-0.064
3PHHHHHH7	0.4111	-	-	-	-	-	-
7PHHHHHH3	0.4394	-	-	-	-	-	-
3PBBBBBB7	0.3802	-	-	-	-	-	-
7PBBBBBB3	0.4060	-	-	-	-	-	-
65PCCCCCCM2	0.5103	No	0.703	30h Mammalian reticulocytes in vitro >20h Yeast in vivo >10h E.coli in vivo	31.20	103	-0.031
65PHHHHHHM2	0.4568	-	-	-	-	-	-
65PBBBBBBM2	0.4433	-	-	-	-	-	-

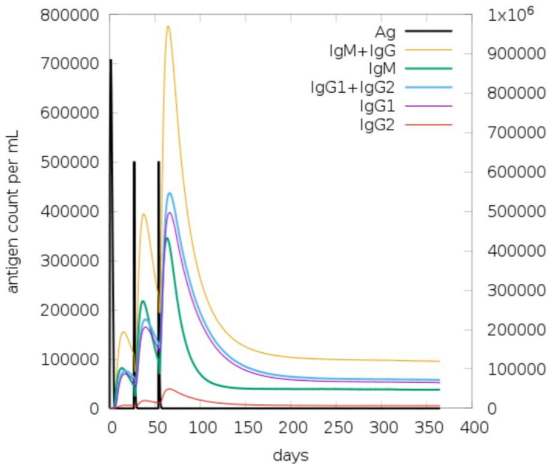

(**a**)

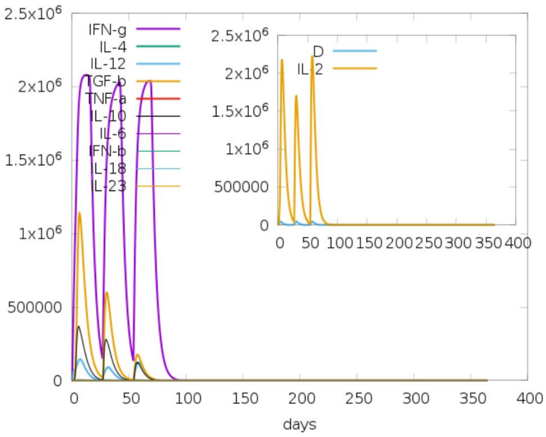

(**b**)

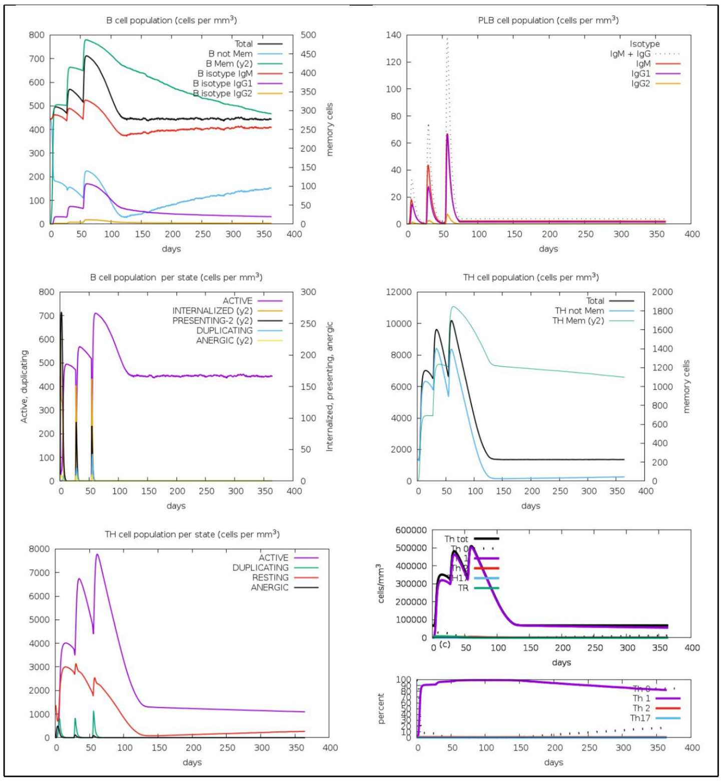

(c)

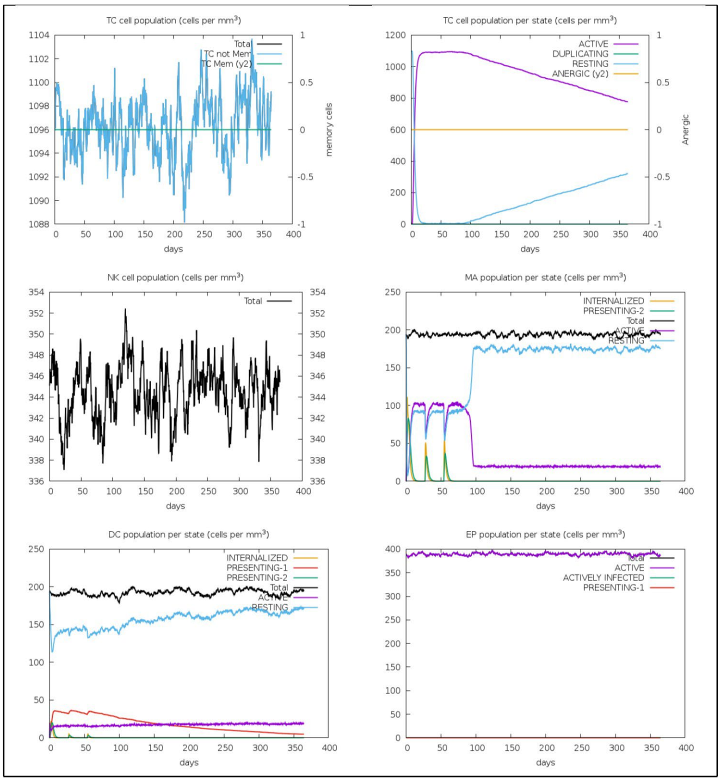

(**d**)

Figure S1. (**a**) Changes in the counts and titers of various immunoglobulins and immune complexes as time goes by after vaccine candidate 7PC12345H12B123 was injected on day 0, 28, and 56. (**b**) Concentration of cytokines and interleukins after injection of vaccine 7PC12345H12B123, and inset plot shows danger signal together with the leukocyte growth factor IL-2. (**c**) The B lymphocytes, plasma B lymphocytes, CD4+ T lymphocytes and T-helper lymphocytes counts shown from vaccine 7PC12345H12B123. (Act=active, Intern = internalized the Ag, Pres II = presenting on MHCII, Dup = in the mitotic cycle, Anergic = anergic, Resting = not active). (**d**) The CD8+ T-cytotoxic lymphocytes, natural killer cells, macrophages, dendritic cells, and epithelial cells counts are shown from vaccine 7PC12345H12B123. (Act =active, Intern = internalized the Ag, PresII = presenting on MHCII, Dup = in the mitotic cycle, Anergic = anergic, Resting = not active).

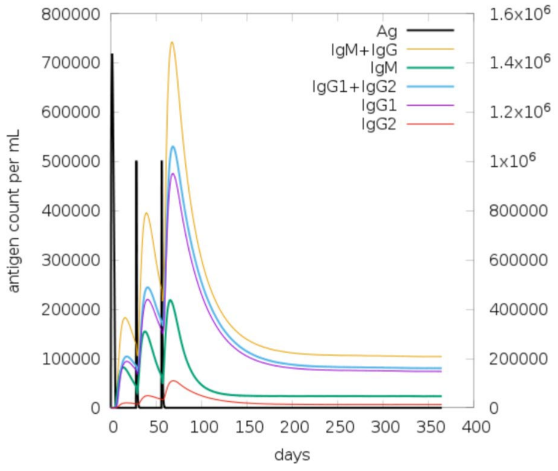

(a)

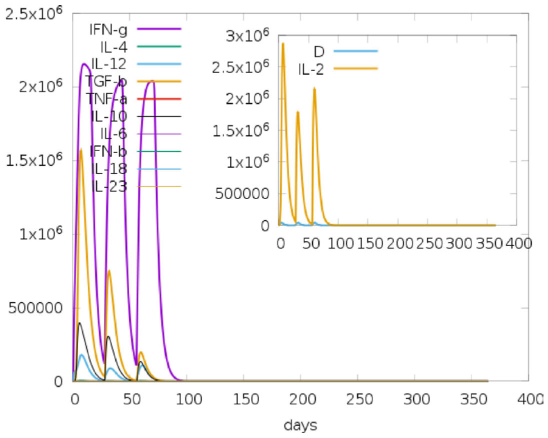

(**b**)

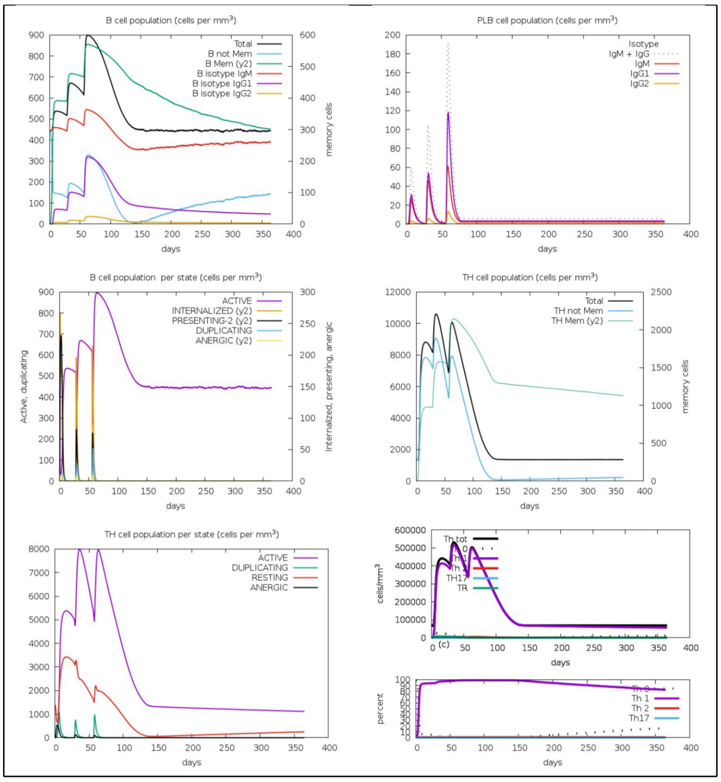

(c)

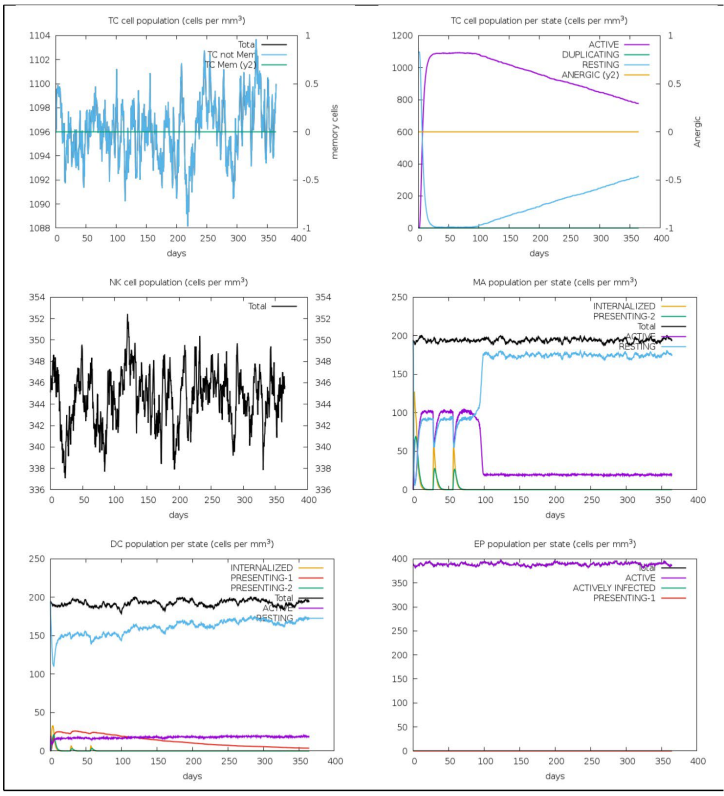

(**d**)

Figure S2. (**a**) Changes in the counts and titers of various immunoglobulins and immune complexes as time goes by after vaccine candidate 7PCHB3 was injected on day 0, 28, and 56. (**b**) Concentration of cytokines and interleukins after injection of vaccine 7PCHB3, and inset plot shows danger signal together with the leukocyte growth factor IL-2. (**c**) The B lymphocytes, plasma B lymphocytes, CD4+ T lymphocytes and T-helper lymphocytes counts shown from vaccine 7PCHB3. (Act=active, Intern = internalized the Ag, Pres II = presenting on MHCII, Dup = in the mitotic cycle, Anergic = anergic, Resting = not active). (**d**) The CD8+ T-cytotoxic lymphocytes, natural killer cells, macrophages, dendritic cells, and epithelial cells counts are shown from vaccine 7PCHB3. (Act =active, Intern = internalized the Ag, PresII = presenting on MHCII, Dup = in the mitotic cycle, Anergic = anergic, Resting = not active).

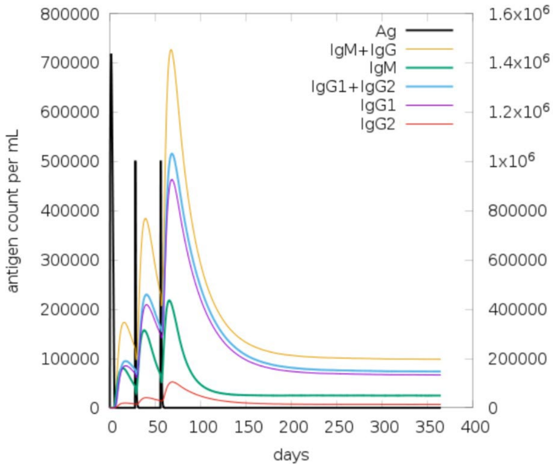

(a)

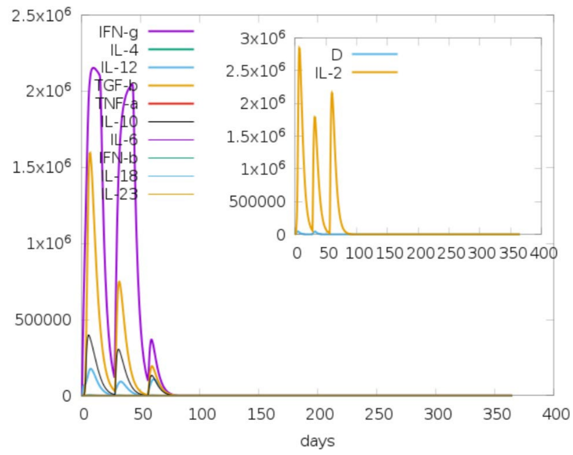

(**b**)

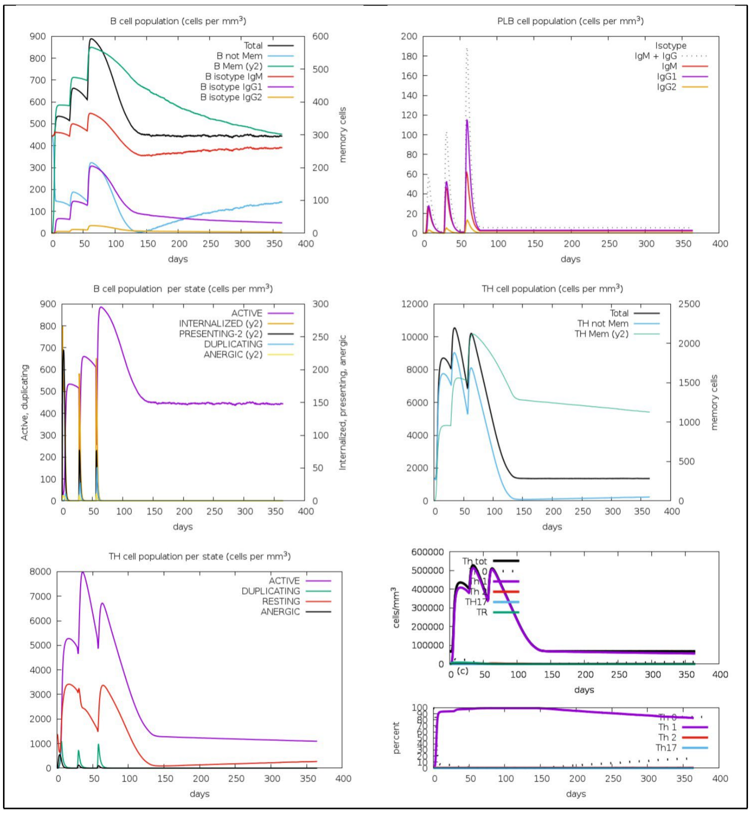

(c)

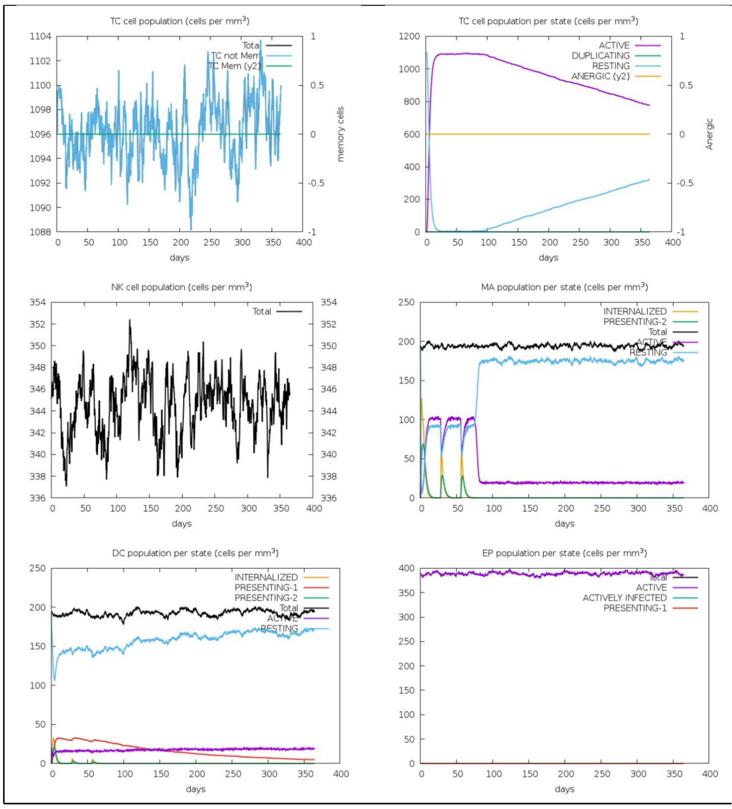

(**d**)

Figure S3. (**a**) Changes in the counts and titers of various immunoglobulins and immune complexes as time goes by after vaccine candidate 7PHBC3 was injected on day 0, 28, and 56. (**b**) Concentration of cytokines and interleukins after injection of vaccine 7PHBC3, and inset plot shows danger signal together with the leukocyte growth factor IL-2. (**c**) The B lymphocytes, plasma B lymphocytes, CD4+ T lymphocytes and T-helper lymphocytes counts shown from vaccine 7PHBC3. (Act=active, Intern = internalized the Ag, Pres II = presenting on MHCII, Dup = in the mitotic cycle, Anergic = anergic, Resting = not active). (**d**) The CD8+ T-cytotoxic lymphocytes, natural killer cells, macrophages, dendritic cells, and epithelial cells counts are shown from vaccine 7PHBC3. (Act =active, Intern = internalized the Ag, PresII = presenting on MHCII, Dup = in the mitotic cycle, Anergic = anergic, Resting = not active).

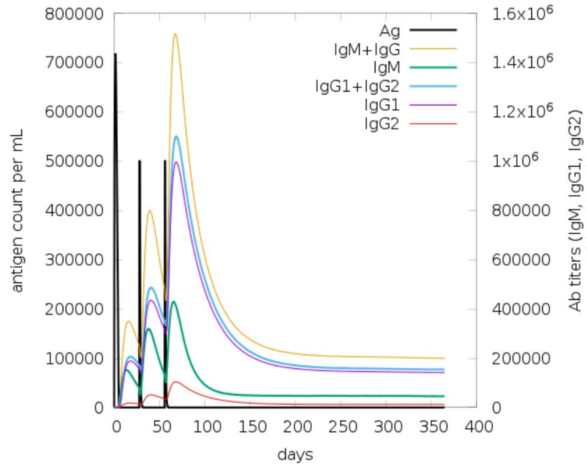

(a)

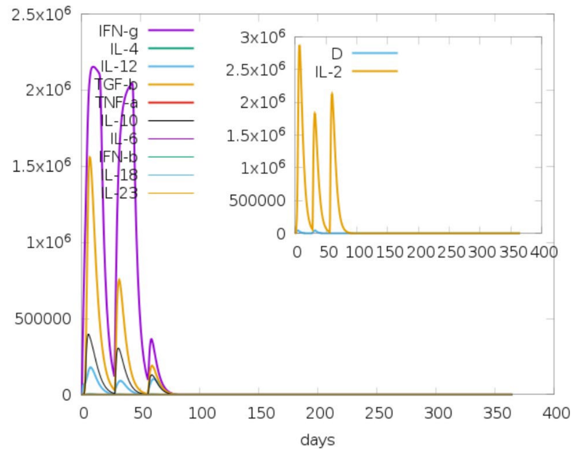

(b)

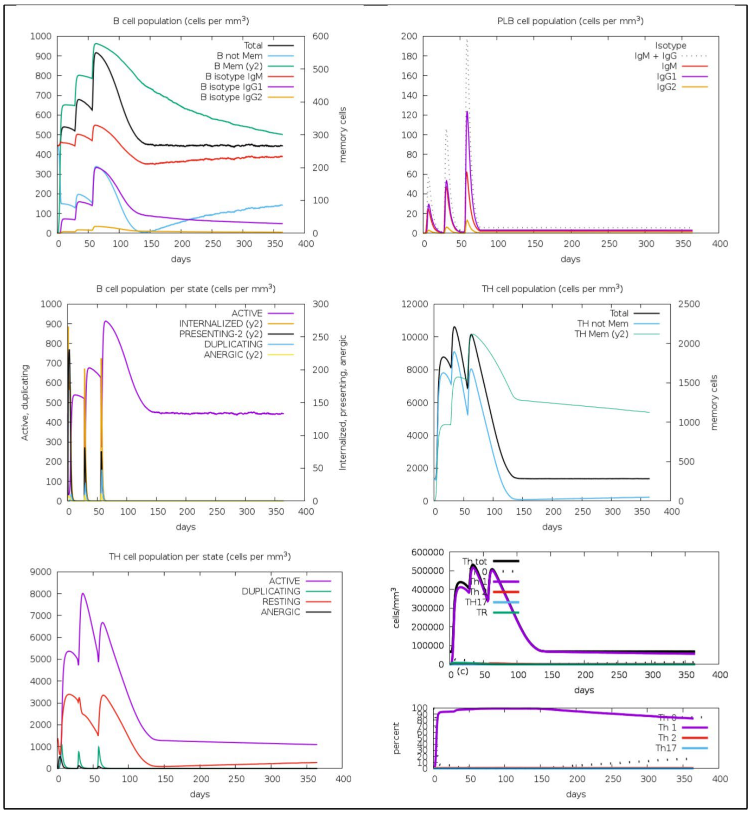

(c)

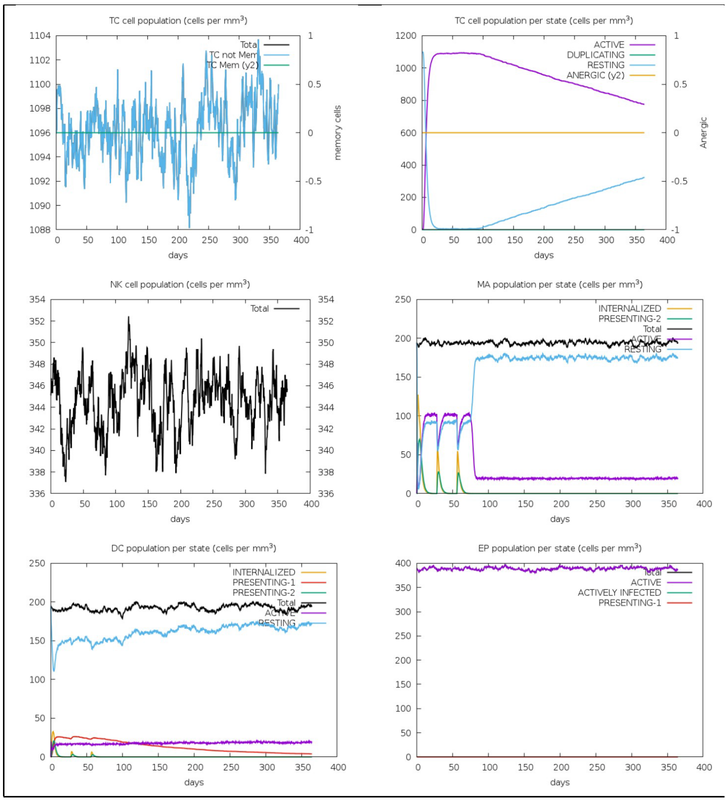

(d)

Figure S4. (**a**) Changes in the counts and titers of various immunoglobulins and immune complexes as time goes by after vaccine candidate 7PBCH3 was injected on day 0, 28, and 56. (**b**) Concentration of cytokines and interleukins after injection of vaccine 7PBCH3, and inset plot shows danger signal together with the leukocyte growth factor IL-2. (**c**) The B lymphocytes, plasma B lymphocytes, CD4+ T lymphocytes and T-helper lymphocytes counts shown from vaccine 7PBCH3. (Act=active, Intern = internalized the Ag, Pres II = presenting on MHCII, Dup = in the mitotic cycle, Anergic = anergic, Resting = not active). (**d**) The CD8+ T-cytotoxic lymphocytes, natural killer cells, macrophages, dendritic cells, and epithelial cells counts are shown from vaccine 7PBCH3. (Act =active, Intern = internalized the Ag, PresII = presenting on MHCII, Dup = in the mitotic cycle, Anergic = anergic, Resting = not active).

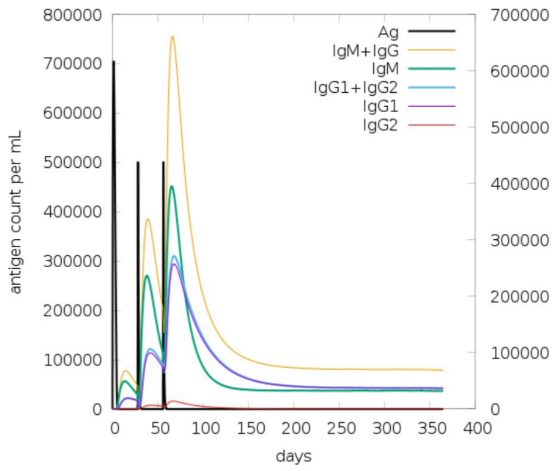

(a)

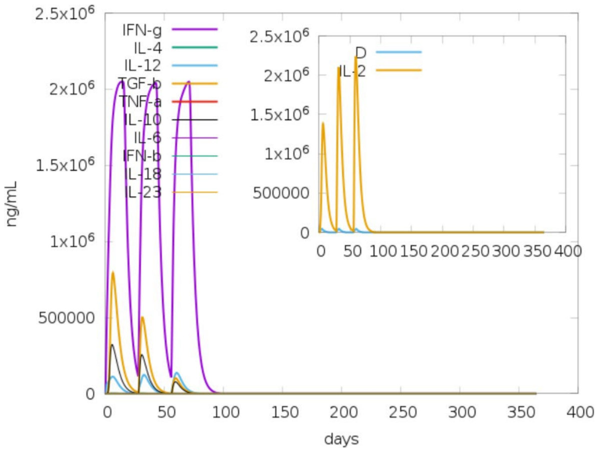

(b)

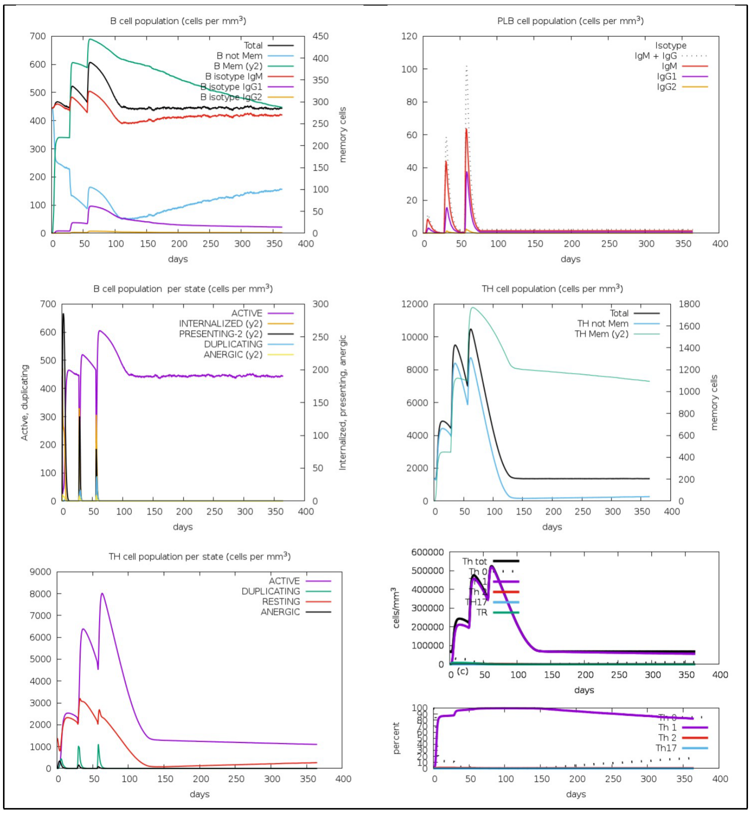

(c)

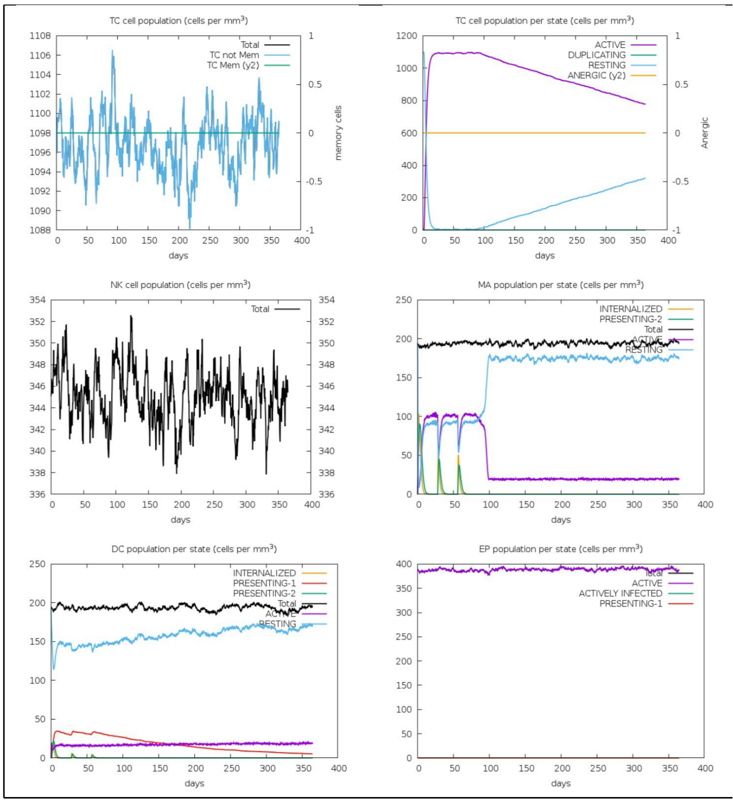

(d)

Figure S5. (**a**) Changes in the counts and titers of various immunoglobulins and immune complexes as time goes by after vaccine candidate 7PCCCCCC3 was injected on day 0, 28, and 56. (**b**) Concentration of cytokines and interleukins after injection of vaccine 7PCCCCCC3, and inset plot shows danger signal together with the leukocyte growth factor IL-2. (**c**) The B lymphocytes, plasma B lymphocytes, CD4+ T lymphocytes and T-helper lymphocytes counts shown from vaccine 7PCCCCCC3. (Act=active, Intern = internalized the Ag, Pres II = presenting on MHCII, Dup = in the mitotic cycle, Anergic = anergic, Resting = not active). (**d**) The CD8+ T-cytotoxic lymphocytes, natural killer cells, macrophages, dendritic cells, and epithelial cells counts are shown from vaccine 7PCCCCCC3. (Act =active, Intern = internalized the Ag, PresII = presenting on MHCII, Dup = in the mitotic cycle, Anergic = anergic, Resting = not active).

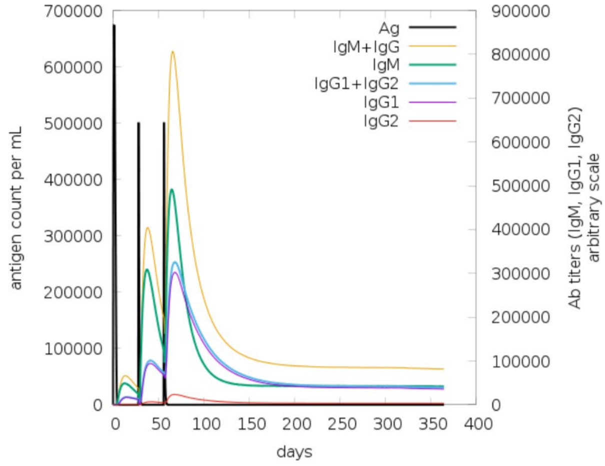

(a)

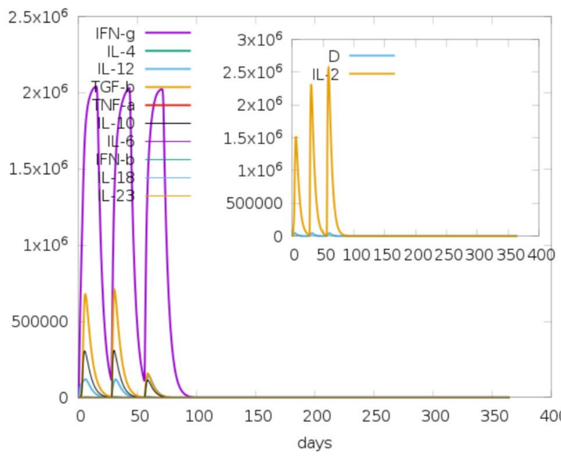

(b)

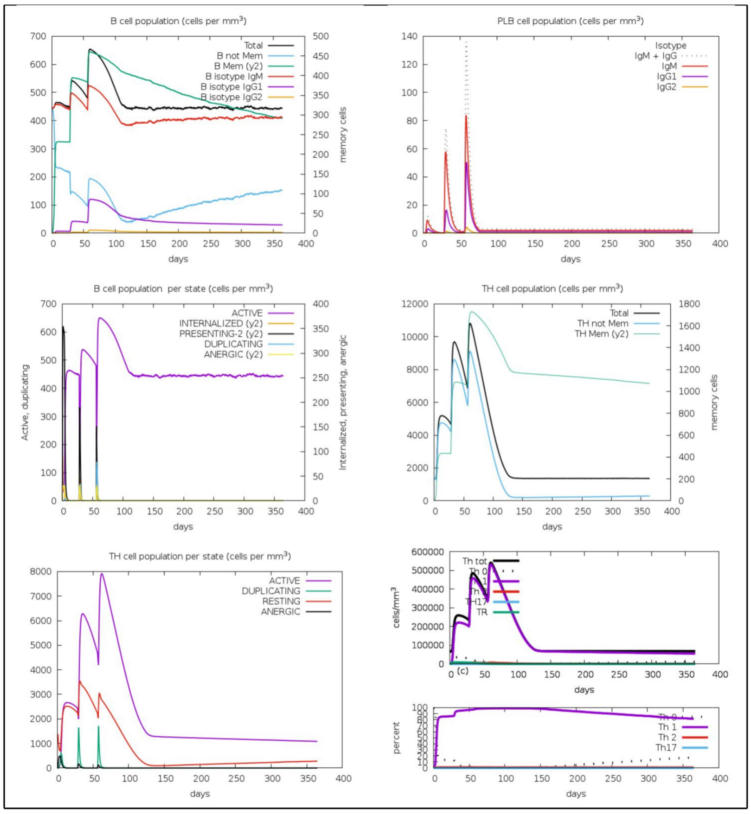

(**c**)

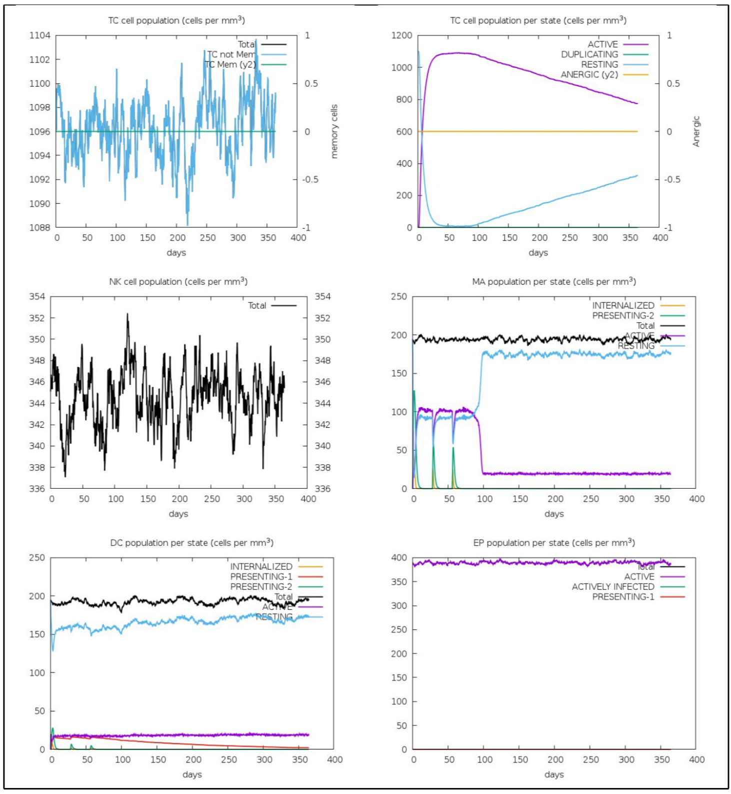

(**d**)

Figure S6. (**a**) Changes in the counts and titers of various immunoglobulins and immune complexes as time goes by after vaccine candidate 65PCCCCCCM2 was injected on day 0, 28, and 56. (**b**) Concentration of cytokines and interleukins after injection of vaccine 65PCCCCCCM2, and inset plot shows danger signal together with the leukocyte growth factor IL-2. (**c**) The B lymphocytes, plasma B lymphocytes, CD4+ T lymphocytes and T-helperlymphocytescountsshown from vaccine 65PCCCCCCM2. (Act=active, Intern = internalized the Ag, Pres II = presenting on MHCII, Dup = in the mitotic cycle, Anergic = anergic, Resting = not active). (**d**) The CD8+ T-cytotoxic lymphocytes, natural killer cells, macrophages, dendritic cells, and epithelial cells counts are shown from vaccine 65PCCCCCCM2. (Act =active, Intern = internalized the Ag, PresII = presenting on MHCII, Dup = in the mitotic cycle, Anergic = anergic, Resting = not active).

Table S7. Binding energies and their components (kcal/mol) of vaccine candidates for TLR3 calculated by the MM/PBSA method.

	**7PC12345H12B123**	**7PCHB3**	**7PCCCCCC3**	**65PCCCCCCM2**
**Van der Waals energy**	-3422.3654	-5627.3792	-5162.6752	-2695.9893
**Electrostatic energy**	-2366.8447	-2789.1733	-2099.9461	643.0187
**Polar solvation energy**	2418.5677	2896.8858	2203.7195	-453.3249
**Non-polar solvation energy**	-99.4619	-209.3514	-200.9752	-172.8320
**Total binding energy**	-3251.9072	-5305.3836	-4834.6317	-2282.2010

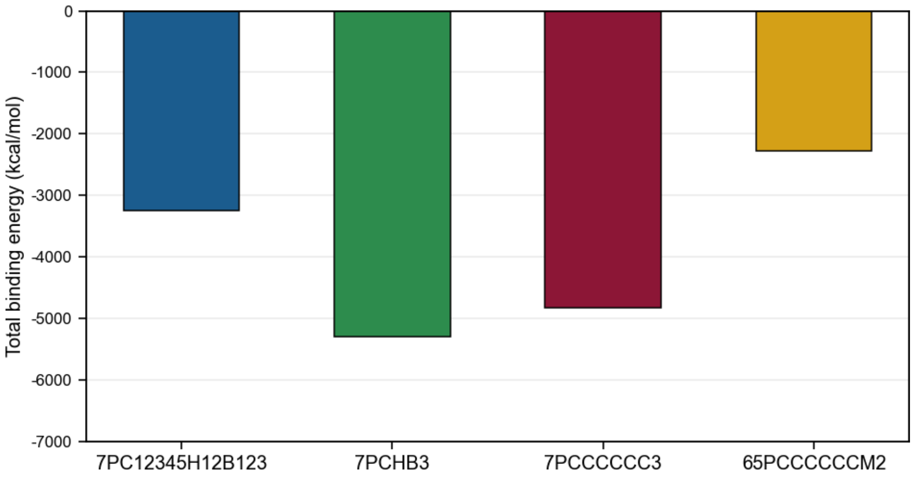

Figure S7. Total binding energies (kcal/mol) of vaccine candidates for TLR3 calculated by MM/PBSA method.

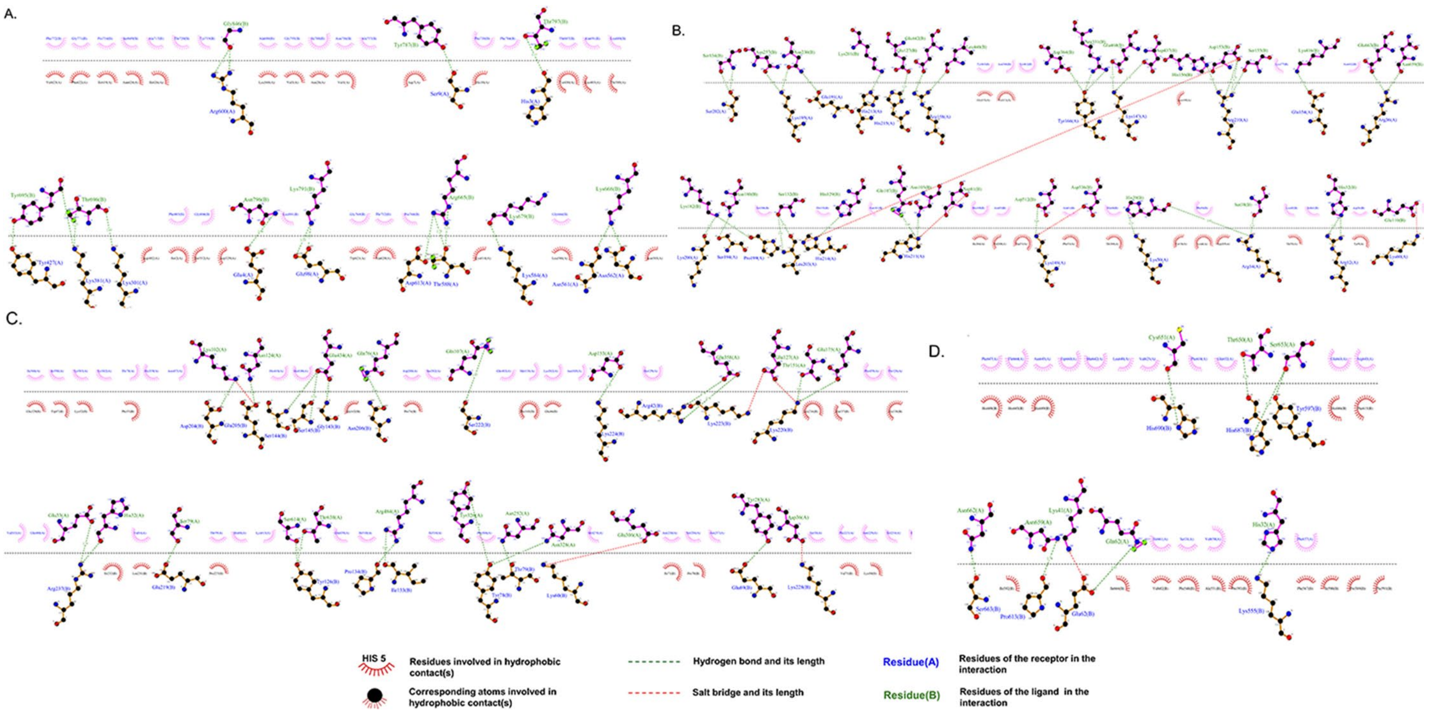

Figure S8. Eyelash diagram of the interaction in vaccines. (**a**) 7PC12345H12B123. (**b**) 7PCHB3. (**c**) 7PCCCCCC3. (**d**) 65PCCCCCCM2.

Table S8. Codon Adaptation Index (CAI) and GC content after codon optimization for each candidate.

	**7PC12345H12B123**	**7PCHB3**	**7PCCCCCC3**	**65PCCCCCCM2**
**Codon Adaptation Index (CAI)**	0.94	0.94	0.94	0.92
**GC content**	53.26%	52.07%	51.43%	56.39%

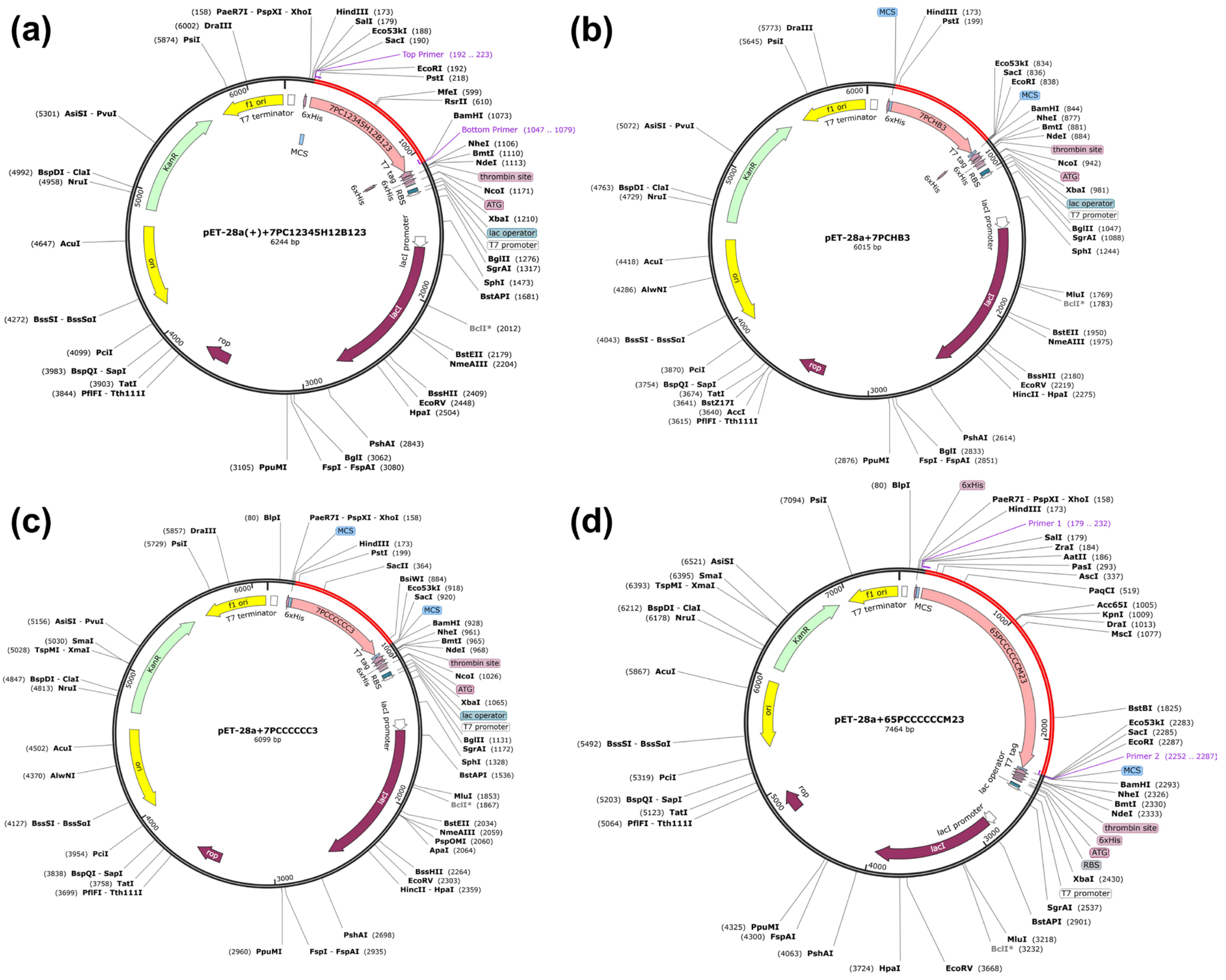

Figure S9. In silico restriction cloning of the final vaccine construct into pET-28a (+) expression vector, where red part represents the vaccine insert and the black circle shows the vector. (**a**) 7PC12345H12B123. (**b**) 7PCHB3. (**c**) 7PCCCCCC3. (**d**) 65PCCCCCCM2.

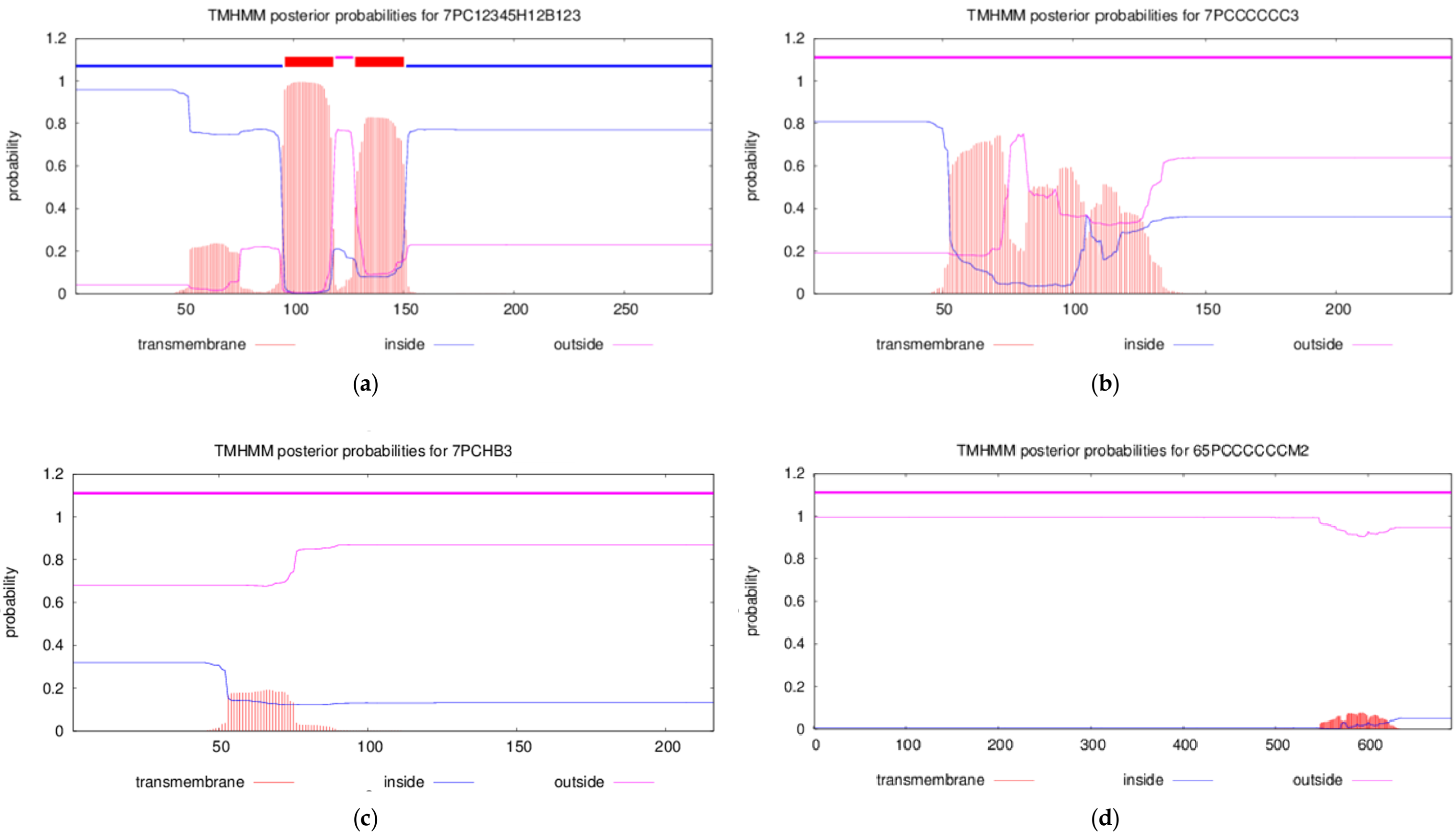

Figure S10. TMHMM prediction of transmembrane helices for plasmid-ready vaccine candidates. (a) NV6 (7PC12345H12B123). (b) NV4 (7PCCCCCC3). (c) NV1 (7PCHB3). (d) NV5 (65PCCCCCCM2).

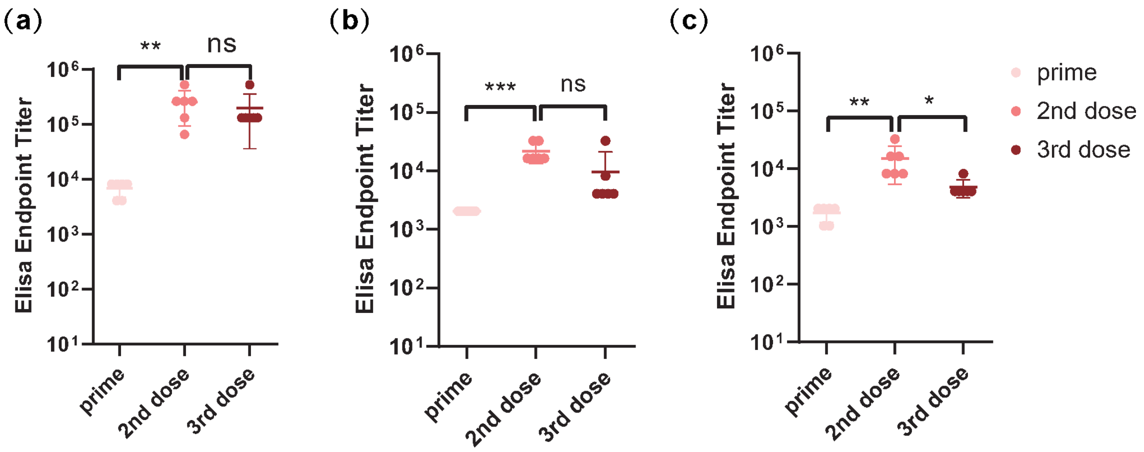

Figure S11. Antibody response in mice immunized with (a) NV5, (b) NV4, and (c) NV1. Complete Freund's adjuvant was used for the first dose, incomplete Freund'sadjuvant for the boosters. ELISA endpoint titers were defined as the reciprocal of the highest serum dilution with absorbance ≥ 0.1 OD above blank. Each symbol denotes one mouse; lines indicate group GMT,data are mean values ± SD, n=6. Statistical significance: ns, $P > 0.05$; *, $P < 0.05$; **, $P < 0.01$; ***, $P < 0.001$.
